# Draft Genome Sequencing of Ascomycetes Yeast *Pichia membranifaciens* KS47-1, Which Shows High Acetate Resistance in Lignocellulosic Feedstock Hydrolysate

**DOI:** 10.1128/genomeA.01672-16

**Published:** 2017-02-23

**Authors:** Masaaki Konishi, Tomoko Arakawa, Yuta Kato, Masashi Ishida, Jun-ichi Horiuchi

**Affiliations:** a Department of Biotechnology and Environmental Chemistry, Kitami Institute of Technology, Kitami, Hokkaido, Japan; b Department of Biotechnology and Environmental Chemistry, Graduate School of Engineering, Kitami Institute of Technology, Kitami, Hokkaido, Japan; c Department of Biomolecular Engineering, Kyoto Institute of Technology, Matsugasaki, Sakyo-ku, Kyoto, Japan

## Abstract

*Pichia membranifaciens* KS47-1 is capable of growing on hydrolysate containing high concentrations of acetate and other growth inhibitors. To reveal the acetate-resistant associate genes of strain KS47-1, we present the 11.4-Mb draft genome sequence.

## GENOME ANNOUNCEMENT

Weak acids, including acetate and formate, are by-products caused during the preparation of hydrolysate of lignocellulosic feedstocks to obtain saccharides from cellulose and hemicellulose for biofuels and biopolymer productions (1). Thermochemical processes, including acid hydrolysis and steam explosion, and enzymatic processes are often considered a most promising approach. Thermochemical treatments usually degrade hemicellulose, leading to the formation of saccharides and inhibitory materials (2). Acetic acid, as one of the inhibitory materials, is formed primarily by hydrolysis of acetyl groups of hemicellulose and is often composed of major aliphatic acids in hydrolysate. A high concentration of acetate is strongly harmful for yeast growth to ferment saccharides to biofuels and other biomaterials (2). Although detoxification has been studied, these detoxifications raise the production cost and are mostly difficult to be applied at the commercial stage. Some molecular biological approaches have been reported (3, 4). These engineered strains have provided inhibitory resistance against the sole material, and they have not proved the resistance against other inhibitors at all.

We have already isolated an acetate-tolerant yeast, *Pichia membranifaciens* KS47-1, which is capable of growing on corncob hydrolysate, which strongly inhibits conventional yeast growth, and a medium containing 400 mM acetate. We are considering that the strain has great potential for producing biofuel and biomaterial from lignocellulosic feedstocks. Therefore, draft genome sequencing was performed to reveal the acetate resistance-associated genes of KS47-1 in the present study.

Draft genome sequencing was performed using the HiSeq 2000 sequencer (Illumina). Total genomic DNA (1 μg) was prepared using the alkaline lysis method using sodium dodecyl sulfate. Fragment library construction and sequencing were carried out by Hokkaido System Sciences. The sequence reads from the paired-end library (400 bp) were initially assembled by Velvet version 1.2.08 using the DDBJ Read Sequence Annotation Pipeline (5). Coding sequences (CDSs), tRNA, and rRNA were detected by AUGUSTUS 2.5.5, tRNAscan-SE 1.23, NCBI BLAST 2.2.18, and RNAmmer 1.2 using the Microbial Genome Annotation Pipeline of DDBJ. Annotation of CDSs was performed by NCBI BLAST 2.2.18 using databases, including RefSeq (release date: 2014/09/11), TrEMBL release 2014_04 (release date: 2014/04/17), and nonredundant databases (release date: 2014/10/01).

Draft sequencing was performed by the Illumina HiSeq system, with a total of 20,493,162 reads. The sequence reads initially assembled into 522 contigs. The *N*_50_ contig size and maximum and minimum contig sizes were 552,197, 552,197, and 121 bp, respectively. The total contig size reached 11,399,492 bp, which was larger than that of *Pichia pastoris*, at 9.43 Mbp (6). The genome was composed of 4,225 putative coding genes or open reading frames (ORFs), four rRNA genes, and 174 tRNA genes.

Acetate tolerance-associated genes were searched on the basis of sequence data of *Saccharomyces cerevisiae* S288c. The aquaglyceroporin gene *FPS1* and acetate-associated transcriptional activator gene *HAA1* were not detected. CDSs with locus tags PMKS-000772, PMKS-000129, and PMKS-00142 were closely related to, H^+^-ATPase, *PMA1*, ATP-dependent permease, *PDA1*, and predicted H^+^-multidrug transporter, TPO2 or TPO3, respectively.

### Accession number(s).

The whole-genome shotgun project has been deposited at DDBJ/EMBL/GenBank under accession numbers BDGI01000001 to BDGI01000522 (as 522 entries).
